# Effects of scoliosis specific conservative management of an adolescent female with is with high risk of progression

**DOI:** 10.1186/1748-7161-9-S1-P6

**Published:** 2014-12-04

**Authors:** Margaret Gogin, Peter Arndt, Cindy Marti

**Affiliations:** 1 Spinal Dynamics of Wisconsin, Wauwatosa, WI, USA

## Background

Daily scoliosis specific exercises have been used in European clinics since the first half of the century for stabilizing scoliotic curves in adolescents. Over the past decade, physiotherapists in the US have begun to use these strategies as well.

## Aim

The aim of this study is to provide a case study of a patient with progressive AIS, and her current outcomes in a US clinic.

## Methods

A 12 y/o female diagnosed with AIS presented to the clinic, with recent history of 6 degree progression (over 3 months)in thoracic and lumbar curves to be 21 degrees right thoracic and 27 degrees left lumbar, Risser 0, premenarchal, at initiation of treatment. Risk of progression, as calculated using Lonstein-Carlson equation, was greater than 90%. In addition, she had a ScoliScore of 188, indicating a “high risk of progression to a severe curve.” (3) She was prescribed a Boston brace to be worn 14 hours per day. She also was trained in scoliosis specific exercises according to SEAS and Schroth, and was compliant with a 20 minute home exercise program, five times per week.

## Results

Over the course of 16 months of treatment and observation, this patient was 1.5 years post menarchal, Risser 0, and has increased in height by 3.25 inches. She patient had experienced a reduction in her curve to be currently 12 degrees right thoracic, and 12 degrees left lumbar as measured by Cobb method. She has also noted improvements posturally as noted by DIERS formetric: coronal imbalance from 11mm L to 0mm, improved kyphosis from 30 to 40 degrees, and lordosis from 24 to 33 degrees , and stable rotation as measured by Nash Moe grade 1.

## Conclusion

The results show that scoliosis specific stabilization exercises can be successful at halting progression over a 16 month period of time in an adolescent with IS with high risk of progression.

## Consent

Written informed consent was obtained from the patient's parents for publication of this Case report. A copy of the written consent is available for review by the Editor of this journal.

